# Decreased Proliferation in the Neurogenic Niche, Disorganized Neuroblast Migration, and Increased Oligodendrogenesis in Adult Netrin-5-Deficient Mice

**DOI:** 10.3389/fnins.2020.570974

**Published:** 2020-11-26

**Authors:** Shunsuke Ikegaya, Yurika Iga, Sumiko Mikawa, Li Zhou, Manabu Abe, Kenji Sakimura, Kohji Sato, Satoru Yamagishi

**Affiliations:** ^1^Department of Organ and Tissue Anatomy, Hamamatsu University School of Medicine, Hamamatsu, Japan; ^2^Department of Cellular Neurobiology, Brain Research Institute, Niigata University, Niigata, Japan; ^3^Center for Coordination of Research Facilities, Institute for Research Promotion, Niigata University, Niigata, Japan; ^4^Department of Animal Model Development, Brain Research Institute, Niigata University, Niigata, Japan

**Keywords:** adult neurogenesis, axon guidance, netrin, subventricular zone, oligodendrogenesis

## Abstract

In the adult mouse brain, neurogenesis occurs mainly in the ventricular-subventricular zone (V-SVZ) and the subgranular zone of the hippocampal dentate gyrus. Neuroblasts generated in the V-SVZ migrate to the olfactory bulb via the rostral migratory stream (RMS) in response to guidance molecules, such as netrin-1. We previously showed that the related netrin-5 (NTN5) is expressed in Mash1-positive transit-amplifying cells and doublecortin-positive neuroblasts in the granule cell layer of the olfactory bulb, the RMS, and the subgranular zone of the adult mouse brain. However, the precise role of NTN5 in adult neurogenesis has not been investigated. In this study, we show that proliferation in the neurogenic niche is impaired in NTN5 knockout mice. The number of proliferating (EdU-labeled) cells in NTN5 KO mice was significantly lower in the V-SVZ, whereas the number of Ki67-positive proliferating cells was unchanged, suggesting a longer cell cycle and decreased cell division in NTN5 KO mice. The number of EdU-labeled cells in the RMS and olfactory bulb was unchanged. By contrast, the numbers of EdU-labeled cells in the cortex, basal ganglia/lateral septal nucleus, and corpus callosum/anterior commissure were increased, which largely represented oligodendrocyte lineage cells. Lastly, we found that chain migration in the RMS of NTN5 KO mice was disorganized. These findings suggest that NTN5 may play important roles in promoting proliferation in the V-SVZ niche, organizing proper chain migration in the RMS, and suppressing oligodendrogenesis in the brain.

## Introduction

Neurogenesis in adult mammals, which occurs mainly in the subgranular zone (SGZ) of the dentate gyrus in the hippocampus and in the ventricular-subventricular zone (V-SVZ), influences learning and memory (Toda and Gage, 2018). In the SGZ, newly generated neurons migrate to the granule cell layer, where they mature and develop dendritic branches. Neuroblasts and transit-amplifying cells generated from neural stem cells (NSCs) and neural precursors in the V-SVZ migrate through the rostral migratory stream (RMS) to the olfactory bulb (OB) (Kaneko et al., 2010; Lim and Alvarez-Buylla, 2016), where they disperse radially and differentiate into granular and periglomerular interneurons in the granule cell layer and glomerular layer, respectively (Sawada et al., 2018). This process is crucial for olfactory function, and Marin et al. (2019) demonstrated that neurogenesis is the mechanism of recovery from olfactory dysfunction induced by excitotoxicity. The increased proliferation of neuroblasts in the V-SVZ contributes to an increase in GABAergic interneurons in the OB related to olfactory function.

Oligodendrocyte precursor cells (OPCs) are generated in the V-SVZ and throughout the parenchyma by local proliferation, migrate extensively along the vasculature, and proliferate at their destinations in the central nervous system (Tsai et al., 2016). This “oligovascular” unit is involved in oligodendrogenesis, creating the myelin-forming cells, and in angiogenesis in ischemia (Wang et al., 2020). In the adult brain, some OPCs that populate the corpus callosum arise from NSCs in the dorsal region of the V-SVZ (Menn et al., 2006). Under normal conditions, only a small number of oligodendrocytes are newly generated from OPCs, and only one-quarter of OPCs differentiate into oligodendrocytes. Nevertheless, the oligodendroglial density in upper cortical layers increases during adulthood (Hughes et al., 2018). Moreover, oligodendrogenesis from NSCs and OPCs is rapidly induced upon demyelination, such as in multiple sclerosis (Frisen, 2016).

Netrin family proteins, conserved from roundworms to mammals, have an impact on neural circuit formation by attracting and repelling axons and regulating cell migration during neural development (Van Raay et al., 1997; Wang et al., 1999; Yin et al., 2000; Nakashiba et al., 2002). Netrins belong to the laminin superfamily of proteins comprising structural components for the basal membranes of tissues. The N-terminal domains of netrins consist of three epidermal growth factor-like repeats, similar to the laminin gamma chain, whereas their C-terminal domains are not homologous to laminins (Kappler et al., 2000). Netrin-1 regulates axon guidance as both attractive and repulsive cues by binding to Unc5 family proteins, deleted in colorectal carcinoma (DCC), neogenin, down syndrome cell adhesion molecule (DSCAM), and integrin family proteins in vertebrates (Arakawa, 2004; Ly et al., 2008; Stanco et al., 2009; Adams and Eichmann, 2010; Castets and Mehlen, 2010; Dun and Parkinson, 2017). Netrin-1 also modulates oligodendrogenesis in various regions. For instance, it repels OPCs migrating into the developing spinal cord and prevents them from entering the retinal end of the developing optic nerve (Spassky et al., 2002; Tsai and Miller, 2002), and netrin-1 overexpression promotes white matter repair and remodeling after focal cerebral ischemia (He et al., 2013).

We previously reported on the structure and expression patterns of netrin-5 (NTN5), which is homologous to netrin-1 (Yamagishi et al., 2015). NTN5 was strongly expressed in Mash1-positive cells and doublecortin (DCX)-positive neuroblasts in the OB, RMS, V-SVZ, and SGZ and also expressed in some vascular endothelial cells in both cerebral cortex and the striatum in adult mice (Yamagishi et al., 2015). Deletion of *Ntn5* from boundary cap cells revealed its contribution to the development of the central/peripheral nervous system boundary and its role in preventing cells from migrating into the ventral roots of the spinal cord (Garrett et al., 2016). In this study, we investigated the phenotype of NTN5 knockout (KO) mice and characterized the functions of NTN5 in adult neurogenesis. We report that proliferation in the neurogenic niche was reduced and the chain migration of neuroblasts in the RMS was disorganized in NTN5 KO mice, whereas the generation of oligodendrocyte lineage cells increased. These findings suggest roles of NTN5 in promoting proliferation in the neurogenic niche, controlling chain migration, and inhibiting oligodendrogenesis in the adult brain.

## Materials and Methods

### Animals

In the study, 3–4 months old NTN5 KO and control (wild type and heterozygote) littermates were used. NTN5 floxed mice were generated as follows. Briefly, two LoxP sequences were inserted to sandwich a 2.5 kb region containing exons 5 and 6, including the C-terminal coding sequence and 3′ UTR of the *Ntn5* gene, and a pgk-neo cassette flanked by Frp sequences was additionally inserted in front of the loxP sequence on the 3′ side in the opposite direction. The targeting vectors were linearized and transfected into the C57BL/6N-derived embryonic stem cell line RENKA (Mishina and Sakimura, 2007). Embryonic stem cell clones with homologous recombination at the targeting site were injected into ICR 8-cell-stage embryos to obtain chimeras. They were subsequently crossed with C57BL/6N females, and offspring with successful germline transmission of the targeted allele were crossed with C57BL/6N mice expressing Flp recombinase to remove the neomycin resistance cassette (Funato et al., 2016). These mice were crossed with Actb-Flp knock-in mice to remove the neomycin cassette via the Flp-FRT system. Then, the mouse line was crossed with the PGK-Cre line, described previously (Lallemand et al., 1998), to delete the floxed region. In these mice, Cre is under the control of the PGK promoter and expressed throughout the body, including germ cells, resulting in NTN5 KO. The mice were genotyped by PCR. The wild-type and floxed alleles were detected as 530-bp and 630-bp PCR products, respectively, using the following primer pair: 5′-AGAGGGTACCCAGCCTATCT and 5′-GGTGAAGACCAGTCCTTCAG. The deletion of the floxed site results in the absence of the PCR product. To confirm the recombination, another PCR reaction was performed with the following primer pair: 5′-AGAGGGTACCCAGCCTATCT and 5′-GAGACTACCGGGCACCTTTG. NTN5 KO mice are fertile with no obvious phenotype, as reported previously (Garrett et al., 2016). All animal experiments were approved by the Animal Research Committee of Hamamatsu University School of Medicine and those of Niigata University and were carried out in accordance with the in-house guidelines for the care and use of laboratory animals of Hamamatsu University School of Medicine and those of Niigata University.

### EdU Labeling and Reaction

Proliferating cells were labeled as described previously (Zeng et al., 2010). Briefly, mice received intraperitoneal injections of EdU (5-ethynyl-2′-deoxyuridine) at 200 mg/kg body weight. After 1 day and 7 days, mice were sacrificed for immunofluorescence analyses as described below. The EdU reaction was carried out for 2 h at room temperature using a solution containing 50 mM Tris (pH 7.4), 150 mM NaCl, 2 mM CuSO_4_, 10 μM Alexa Fluor azides, and 10 mM sodium ascorbate (added last).

### Histological Analysis

Immunostaining was performed as previously reported (Yamagishi et al., 2019). Briefly, the mice were deeply anesthetized and intracardially perfused with 4% paraformaldehyde in phosphate-buffered saline (PFA/PBS) for 5 min. Brains were dissected, postfixed in 4% PFA/PBS for 10 min, and subsequently immersed in 15 and 30% sucrose/PBS for cryoprotection. After the brains were embedded in optimal cutting temperature compound and frozen at −80°C, sagittal and coronal sections at 20 μm thickness were prepared using a cryostat and stored at −20°C until use. Before staining, the sections were dried, further fixed in 4% PFA/PBS for 10 min, washed with PBS, and permeabilized in 0.3% Triton X-100/PBS for 3 min. Then, the sections were incubated with blocking solution containing 10% donkey serum in 0.1% Triton X-100/PBS for 1 h at room temperature, followed by incubation with primary antibodies in 10% donkey serum/0.1% Triton X-100/PBS overnight at 4°C. The sections were then incubated with Alexa Fluor dye-conjugated secondary antibodies for 30 min at room temperature, followed by nuclear staining with DAPI. The sections were observed, and the images were obtained with a confocal microscope (TCS SP8; Leica, Wetzlar, Germany) in sequential scanning mode for multichannel imaging and with an epifluorescence microscope (IX83; Olympus, Tokyo, Japan) using a tiling scanning function. For hematoxylin and eosin (HE) and Luxol fast blue (LFB) staining, fixed brains were embedded in paraffin, cut into 3-μm-thick sections, and stained with HE or LFB according to standard protocols. The images were obtained with a light microscope (Eclipse E600; Nikon, Tokyo, Japan).

### Antibodies

The following primary antibodies were used for immunohistochemistry: rabbit anti-active caspase-3 (1:500; Abcam, Cambridge, United Kingdom), rabbit anti-DCX (1:500; Cell Signaling Technology, Danvers, MA), rabbit anti-glial fibrillary acidic protein (GFAP) (1:500; Agilent, Santa Clara, CA), goat anti-Olig2 (1:50; R&D Systems, Minneapolis, MN), rabbit anti-Iba1 (1:500; Fuji-Wako, Osaka, Japan), rabbit anti-Ki67 (1:500; Abcam), goat anti-MBP (1:500; Santa Cruz Biotechnology, Dallas, TX), rat anti-MBP (1:1,000; Abcam), mouse anti-NeuN (1:100; Merck-Millipore, Burlington, MA), and rat anti-NG2 (1:500; Abcam). The conjugated secondary antibodies were donkey anti-rabbit, anti-rat, anti-mouse, and anti-goat antibodies (Alexa Fluor 488, 568, and 647; Thermo Fisher Scientific, Waltham, MA) at a dilution of 1:500. For the EdU reaction, azide-modified dyes (Alexa Fluor 488 and 594 azide; Thermo Fisher Scientific) were used.

### Quantifications and Statistical Analysis

To quantify proliferated cells, EdU-labeled cells in the OB, RMS, and V-SVZ were counted in three coronal sections for each mouse under an epifluorescence microscope (Eclipse E600) or a confocal microscope (TCS SP8) by capturing 3D images at a 10-μm thickness. Data are presented as means ± standard errors (SEs). Statistical significance was determined by two-sided unpaired Student’s *t*-tests and a *p*-value of <0.05.

For the analysis of migrating cells in the RMS, images of anti-DCX antibody staining were binarized by ImageJ software. Then, a non-linear regression for Gaussian fit was performed by a least-squares fit using Excel with the “Solver” add in. The reliability of the model was validated by evaluating the residuals plot, replicate test, plausibility of best-fit values, *R*^2^ values, and sum-of-square values. Statistical significance for the Gaussian fit was determined using the F test (Yamagishi et al., 2011).

## Results

### Reduced Cell Proliferation in the V-SVZ of NTN5 KO Mice

**FIGURE 1:**
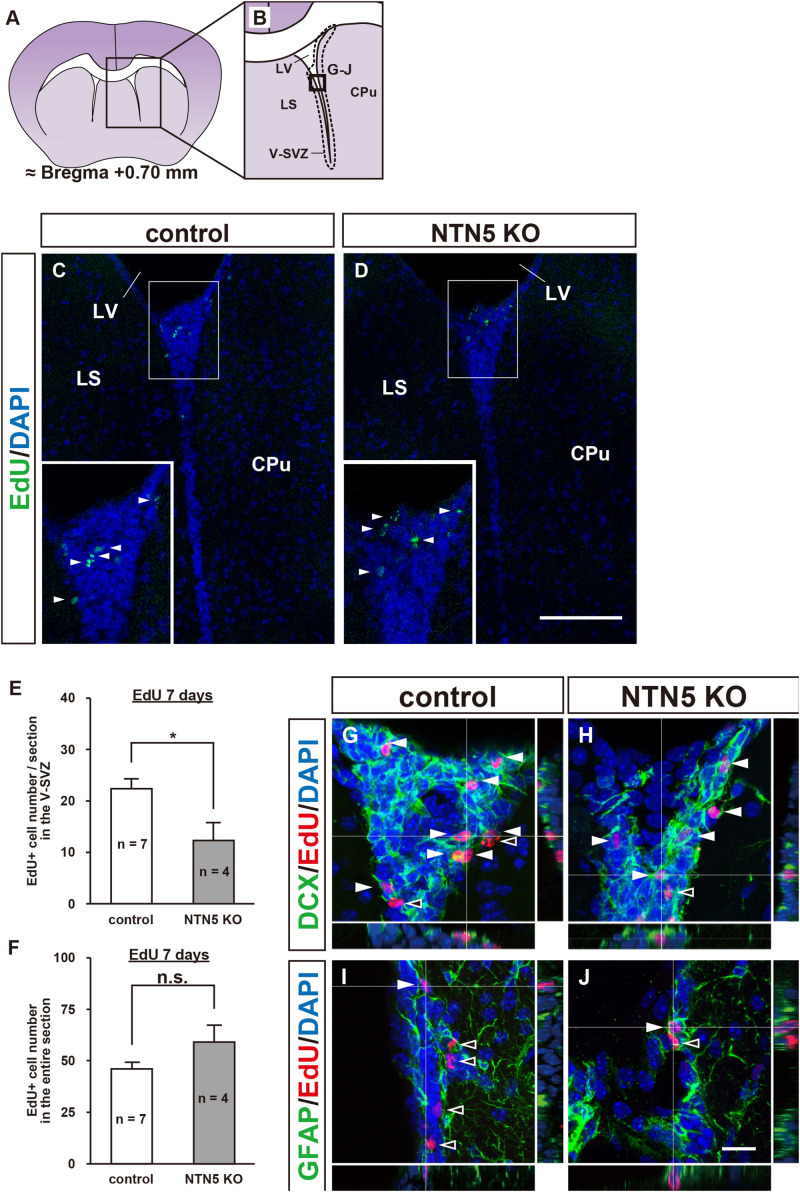
Altered population of EdU-labeled/DCX-positive cells in the V-SVZ of adult NTN5 KO mice 7 days after EdU injection. **(A,B)** Schematic drawing of a coronal section of adult mouse brain at bregma + 0.70 mm. **(B)** Enlargement of boxed region shown in panel **(A)**. Dashed lines indicate a representative area for quantification of the EdU-labeled cells. The square box indicates the area observed for immunostaining. **(C,D)** Representative images of EdU staining of the V-SVZ from control **(C)** and NTN5 KO **(D)** mice 7 days after EdU administration. Insets show higher magnification and EdU-labeled cells (arrowheads). **(E)** Quantification of EdU-labeled cells in the V-SVZ. Three sections per animal were analyzed (*n* = 7 for control, *n* = 4 for NTN5 KO). Data are means ± SEs. *p < 0.05. **(F)** Quantification of EdU-labeled cells in the entire coronal section of the brain in control and NTN5 KO mice. **(G,H)** Representative confocal images of immunostaining with orthogonal views for DCX (green), EdU (red), and DAPI (blue). Most of the EdU-labeled cells observed in the V-SVZ of both control and NTN5 KO mice were DCX positive (white arrowheads). **(I,J)** Representative confocal images with orthogonal views for GFAP (green), EdU (red), and DAPI (blue). Some EdU-labeled cells colocalized with GFAP (white arrowheads). Other EdU-labeled cells were GFAP negative (black arrowheads). Scale bars indicate 100 μm **(C,D)** and 15 μm **(G–J)**. CPu, caudate putamen; LS, lateral septal nucleus; LV, lateral ventricle; V-SVZ, ventricular-subventricular zone.

To investigate the role of NTN5 in immature cell populations in the adult mammalian brain, 3–4 months old NTN5 KO mice and their littermate controls were administrated EdU to label proliferating cells, as NTN5 is expressed in type A and type C cells in the V-SVZ (Yamagishi et al., 2015). Seven days later, the numbers of EdU-labeled cells in the V-SVZ were compared (Figures 1A–D). The number of EdU-labeled cells was decreased to 55% in NTN5 KO mice than in control mice (12.3 ± 3.5 vs. 22.3 ± 2.1 cells/section, respectively, *p* < 0.05) (Figure 1E). However, there was no difference between groups when all of the EdU-labeled cells in coronal sections were counted (Figure 1F). These results suggest that proliferation in the neurogenic niche was inhibited or that proliferated cells rapidly migrated from the V-SVZ in adult NTN5 KO mice.

Next, we characterized the types of cells that were EdU positive in the V-SVZ and found that more than half expressed DCX in both control and NTN5 KO mice (Figures 1G,H). By contrast, some DCX-negative cells were GFAP positive, but none were Olig2, Iba1, or NeuN positive (Figures 1I,J and Supplementary Figures 1C–H; Lazarov et al., 2010; Ming and Song, 2011).

### Increased Production of OPCs in Other Brain Regions of NTN5 KO Mice

**FIGURE 2:**
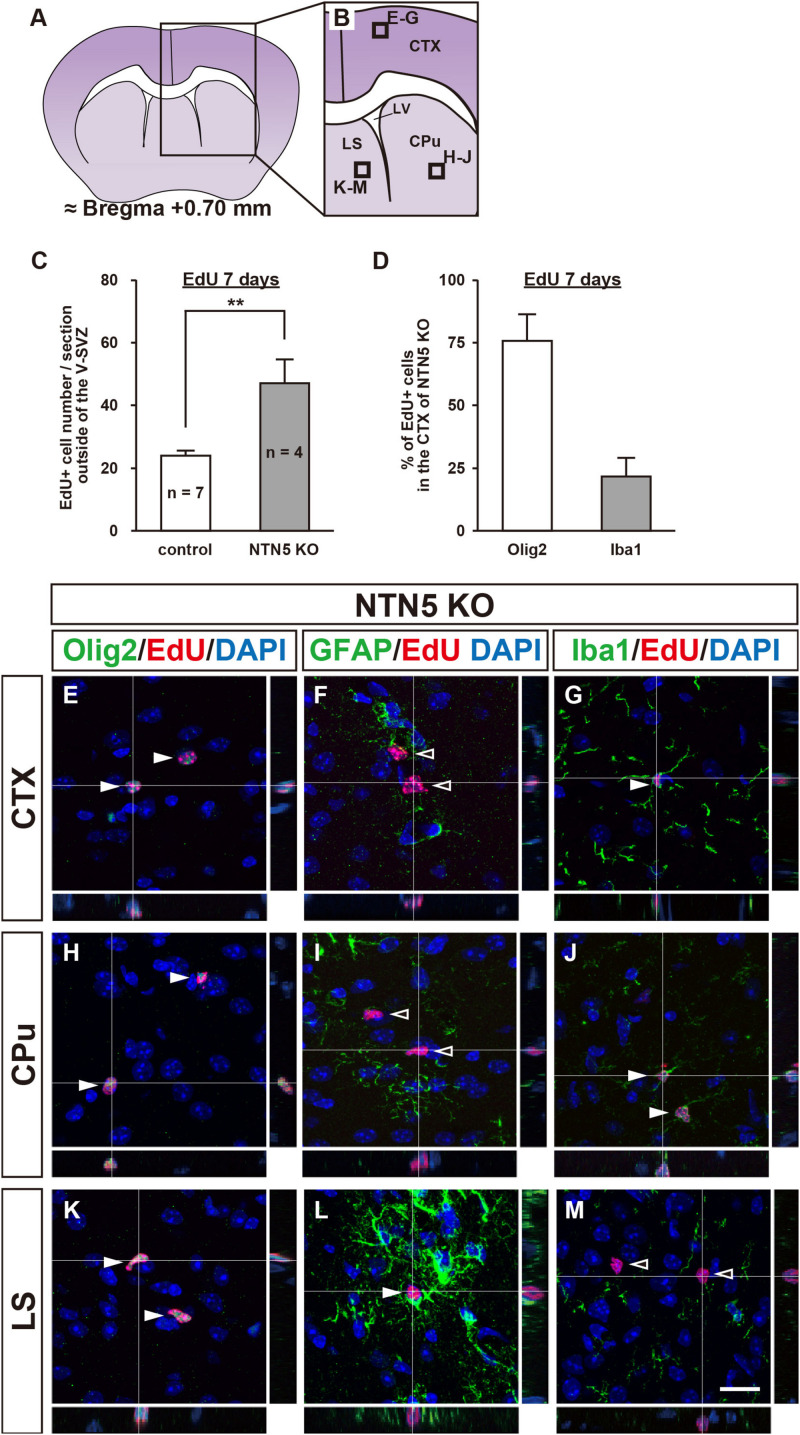
Altered distribution of EdU-labeled cells outside the V-SVZ in NTN5 KO mice 7 days after EdU injection. **(A,B)** Schematic drawings of a coronal section of adult mouse brain at bregma + 0.70 mm. **(C)** Quantification of EdU-labeled cells outside the V-SVZ, namely in the cortex (CTX), caudate putamen (CPu), lateral septal nucleus (LS), corpus callosum, and anterior commissure in coronal brain sections of control and NTN5 KO mice. Three sections per animal were analyzed (*n* = 7 for control, *n* = 4 for NTN5 KO). Data are means ± SEs. **p < 0.01. **(D)** Most EdU-labeled cells were Olig2 positive at 7 days after EdU injection. **(E–G)** Representative confocal images with orthogonal views of the CTX. Sections were stained for EdU (red), DAPI (blue), Olig2 (**E**, green), GFAP (**F**, green), or Iba1 (**G**, green). Almost all EdU-labeled cells observed in the CTX of NTN5 KO mice were Olig2 positive (**E**, white arrowheads). Several EdU-labeled cells colocalized with Iba1 (**G**, white arrowhead). By contrast, there were no GFAP-positive/EdU-labeled cells (**F**, black arrowheads). **(H–J)** Representative confocal images with orthogonal views of the CPu. The main population of EdU-labeled cells was Olig2 positive (**H**, white arrowheads). None of the EdU-labeled cells were GFAP positive in the CPu (**I**, black arrowheads). Several EdU-labeled cells colocalized with Iba1 (**J**, white arrowheads). **(K–M)** Representative confocal images with orthogonal views of a coronal section in the LS. The main population of EdU-labeled cells was Olig2 positive (**K**, white arrowheads). Several EdU-labeled cells colocalized with GFAP (**L**, white arrowhead). There were no EdU-labeled cells that were Iba1 positive in the LS (**M**, black arrowheads). Scale bar indicates 15 μm. LV, lateral ventricle; V-SVZ, ventricular-subventricular zone.

As the number of proliferating cells in NTN5 KO mice was lower in the V-SVZ but not different overall, we carefully counted EdU-labeled cells in areas outside the V-SVZ. Almost all of the EdU-labeled cells in the cortex (CTX), basal ganglia (BG), and lateral septal nucleus (LS) were DCX negative in both control and NTN5 KO mice, and the number of EdU-labeled cells outside the V-SVZ in NTN5 KO mice was significantly higher than in control mice (47.0 ± 7.8 vs. 23.9 ± 1.8 cells/section, respectively, *p* < 0.01) (Figures 2A–C). Furthermore, this increase was significant in the CTX, BG/LS, and in white matter (corpus callosum and anterior commissure) (Table 1).

**TABLE 1:** Increased cell proliferation in NTN5 KO.

**Region^*a*^**	**No. of cells^*b*^**		***p*-value**
	**Control**	**NTN5 KO**	
CTX	9.6 ± 1.1	19.8 ± 3.4	0.012
BG/LS	13.1 ± 1.0	23.8 ± 3.9	0.014
CC/AC	1.0 ± 0.3	3.2 ± 0.6	0.010

*^*a*^AC, anterior commissure; CC, corpus callosum; BG; basal ganglia; CTX, cerebral cortex; LS, lateral septal nucleus.^*b*^Number of EdU-labeled cells proliferating 7 days after EdU injection (n = 7 for control, n = 4 for NTN5 KO). Data are means ± SEs.*

Consistent with a previous study showing that 74% of BrdU-positive cells were NG2-positive OPCs (Dawson, 2003), further analyses revealed that the majority of these EdU-labeled cells were positive for Olig2 (Figure 2D and Supplementary Figure 2C). In the CTX, EdU-labeled cells were mostly Olig2 positive (75.9% ± 10.5%) with some Iba1 positive (21.6% ± 7.4%), but none were GFAP positive (Figures 2E–G). Notably, none of EdU-labeled cells were positive for MBP, a marker for mature oligodendrocytes (Supplementary Figure 2D). In the caudate putamen (CPu; part of the BG), EdU-labeled cells were Olig2 or Iba1 positive but not GFAP positive (Figures 2H–J), whereas GFAP- and Olig2-positive EdU-labeled cells were found in the LS, but none were positive for Iba1 (Figures 2K–M). Together, these data suggest that the majority of newly proliferated cells outside the V-SVZ were oligodendrocyte lineage cells. As NTN5 KO mice had significantly higher numbers of these cells, NTN5 may be secreted from endothelial cells to inhibit oligodendrocyte generation (Yamagishi et al., 2015).

Next, we asked whether the increase of OPCs in NTN5 KO mice would result in morphological changes in white matter. However, we did not find any morphological abnormality by HE, LFB, or anti-MBP staining in NTN5 KO mice (Supplementary Figure 3).

### Number of EdU-Labeled Neuroblasts in the OB Granule Layer Does Not Change in NTN5 KO Mice

**FIGURE 3:**
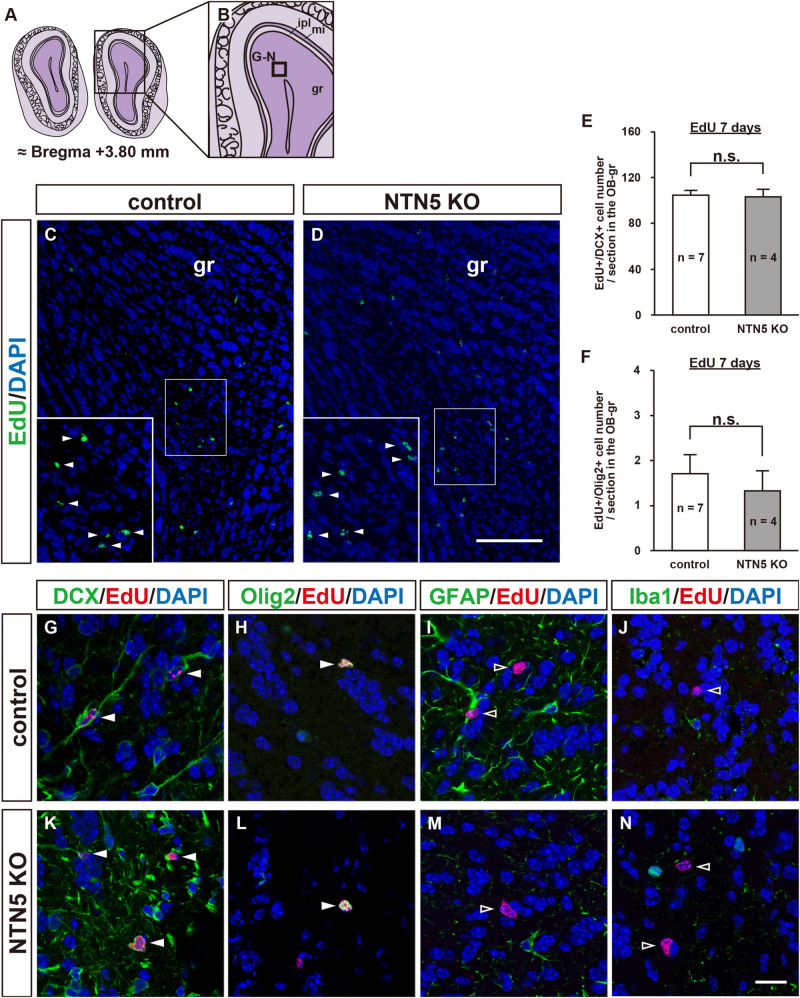
Normal number of EdU-labeled cells in the OB of adult NTN5 KO mice 7 days after EdU injection. **(A,B)** Schematic drawings of the OB of adult mouse brain at bregma + 3.80 mm. **(C,D)** Representative images of EdU staining of granule layer of the OB from control **(C)** and NTN5 KO **(D)** mice 7 days after EdU administration. Insets show higher magnification and EdU-labeled cells (arrowheads). **(E,F)** Quantification of EdU-labeled/DCX-positive cells **(E)** and EdU-labeled/Olig2-positive cells **(F)** in the granule layer of the OB. Three sections per animal were analyzed (*n* = 7 for control, *n* = 4 for NTN5 KO). Data are means ± SEs. n.s., not significant. **(G,K)** Representative confocal images of the OB granule layer immunostained for DCX (green), EdU (red), and DAPI (blue). Almost all EdU-labeled cells in the granule layers of control and NTN5 KO mice were DCX positive (white arrowheads). **(H,L)** Representative confocal images for Olig2 (green), EdU (red), and DAPI (blue). Olig2 staining of the granule layer revealed that there were a few Olig2-positive/EdU-labeled cells (white arrowheads). **(I,M)** Representative confocal images for GFAP (green), EdU (red), and DAPI (blue). GFAP staining of the granule layer revealed that no EdU-labeled cells were GFAP positive (black arrowheads). **(J,N)** Representative confocal images for Iba1 (green), EdU (red), and DAPI (blue). None of the EdU-labeled cells were Iba1 positive in the granule layer (black arrowheads). Scale bar indicates 15 μm. gr, granule layer; ipl, inner plexiform layer; mi, mitral layer.

As NTN5 is expressed by neuroblasts generated in the V-SVZ that migrate via the RMS toward the OB (Yamagishi et al., 2015), we hypothesized that the reduction in proliferative cells observed in NTN5 KO mice would result in fewer cells that migrate to the granule layer of the OB. However, there was no significant difference in the number of EdU-labeled cells in the granule layer of the OB between control and NTN5 KO mice (Figures 3A–E). Olig2-positive cells comprised ∼2% of the EdU-labeled cells in the OB granule layer, and the numbers of these were not significantly different between control and NTN5 KO mice (Figure 3F). Almost all (>98%) of the EdU-labeled cells in the granule layer were DCX positive in control and NTN5 KO mice (Figures 3G,K), suggesting that the supply of newly generated neuroblasts from the V-SVZ/RMS to the OB was maintained in NTN5 KO mice despite the reduced proliferation in the neurogenic niche. The few DCX-negative EdU-labeled cells were Olig2 positive but GFAP and Iba1 negative (Figures 3H–J,L–N).

### Reduced Proliferation in the V-SVZ of NTN5 KO Mice 1 Day After EdU Administration

**FIGURE 4:**
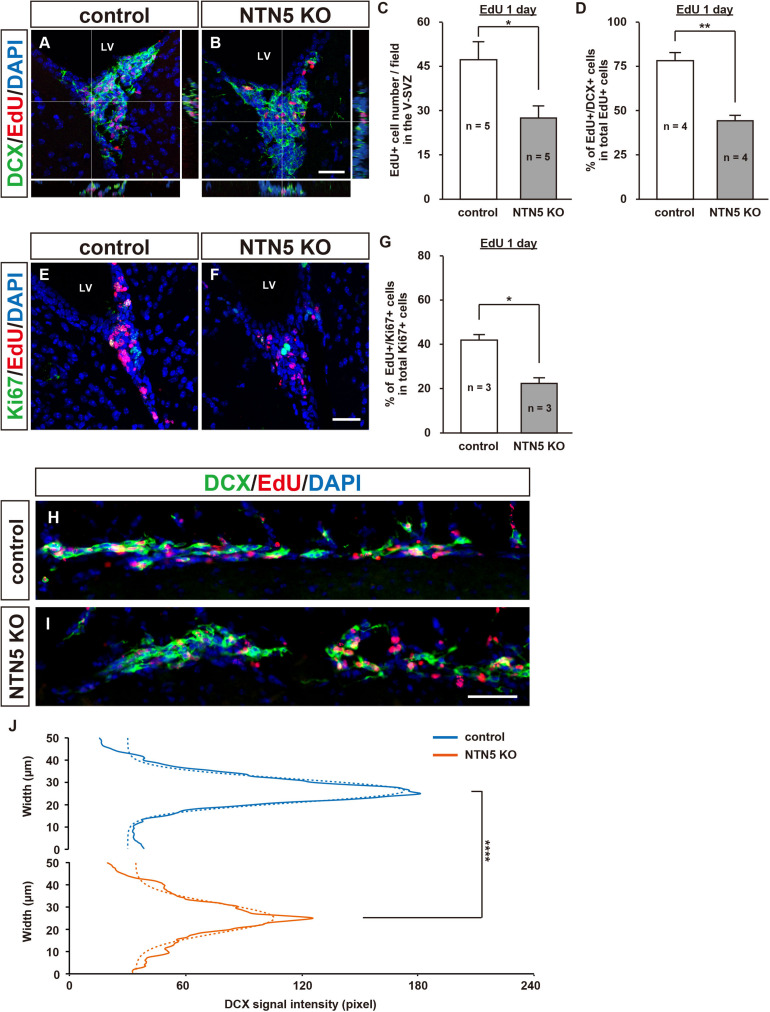
Altered population of EdU-labeled cells in the V-SVZ and misaligned migrating cells in the RMS of adult NTN5 KO mice 1 day after EdU injection. **(A,B)** Representative confocal images with orthogonal views of EdU- and DAPI-labeled sections of the V-SVZ 1 day after EdU administration. Note that there were more EdU-labeled cells than at 7 days after injection (cf. Figures 1C,D). **(C)** Quantification of EdU-labeled cells 1 day after EdU administration. Three fields per animal were analyzed (*n* = 5 for both genotypes). Data are means ± SEs. **(D)** Quantification of DCX-positive cells among EdU-labeled cells. Two fields per animal were analyzed (*n* = 4 for both genotypes). Data are means ± SEs. **(E,F)** Representative images of Ki67-, EdU-, and DAPI-labeled sections of the V-SVZ 1 day after EdU administration. **(G)** Quantification of EdU-labeled cells among Ki67-positive cells in the V-SVZ. Three fields per animal were analyzed (*n* = 3 for both genotypes). Data are means ± SEs. **(H,I)** DCX (green), EdU (red), and DAPI (blue) labeling of sagittal sections (100 μm × 500 μm) of the RMS revealed a broadened distribution of cells migrating to the OB in NTN5 KO mice. **(J)** The distribution of DCX-positive cells in the RMS of NTN5 KO mice was significantly broadened (*p* < 0.0001) according to a Gaussian fitting analysis. Scale bars indicate 30 μm **(A,B,E,F)** and 50 μm **(H,I)**. *p < 0.05, **p < 0.01, ****p < 0.0001. n.s., not significant; LV, lateral ventricle.

To determine whether the decrease in EdU-labeled cells after 7 days in the V-SVZ of NTN5 KO mice (cf. Figure 1E) was a result of increased migration to other brain regions or reduced proliferation, we quantified cell proliferation in the V-SVZ at 1 day after EdU administration (Figure 4). At this time point, the number of EdU-labeled cells was similarly lower in NTN5 KO mice than in control mice (27.5 ± 3.9 vs. 47.0 ± 6.2 cells/field, respectively, Figures 4A–C). This result indicates that the number of proliferated cells in the V-SVZ of NTN5 KO mice is constitutively lower than in control mice. Of note, the density of EdU-labeled cells in the V-SVZ after 1 day was much higher than after 7 days in both control and NTN5 KO mice (cf. Figures 1C,D). The percentage of EdU-labeled cells that were DCX positive was reduced in the NTN5 KO mice (Figure 4D). The reduction of EdU-labeled cells in the V-SVZ of NTN5 KO mice was specific, because we did not see any difference in the SGZ of the hippocampal dentate gyrus after pulse EdU labeling at 1 and 7 days between control and NTN5 KO mice (Supplementary Figure 4). Next, we used Ki67 antibodies to ask whether the number of proliferating cells was reduced in NTN5 KO mice (Figures 4E,F). Unexpectedly, the number of Ki67-positive cells and the cell cycle exit index (percentage of EdU-positive cells that were Ki67 negative) were not significantly different (Supplementary Figures 5A,B). However, the percentage of Ki67-positive cells that were EdU labeled was reduced in the NTN5 KO mice (Figure 4G), suggesting a longer cell cycle. On the other hand, no apoptotic cells (cleaved-caspase-3 positive) were observed (Supplementary Figures 5C,D). Next, we analyzed chain migration in the RMS, which revealed sparsely distributed migrating neuroblasts and a disorganized cellular bundle (Figures 4H–J), though the number of EdU-labeled cells in the RMS were not significantly changed (Supplementary Figure 5F). To analyze the distribution of migrating cells in the RMS, we modeled the DCX signal intensity to a Gaussian fit by non-linear regression (see section “Materials and Methods”). The analysis revealed that the Gaussian fit was dispersed and the peak was shifted down by approximately 60 pixels (39.3%) in NTN5 KO mice compared with that in controls (Figure 4J, *n* = 3 mice per group, *p* < 0.0001, *F*-test), indicating that NTN5 is involved in organizing proper chain-formed migration. By contrast, from the rostral part of the RMS to its termination in the OB, no disorganization was observed, and the density of EdU-labeled cells was normal (Supplementary Figure 6). These results suggest that there may be a compensatory mechanism to replenish the appropriate number of new neurons in the granule layer of the OB in the mutant mice.

## Discussion

In this study, we showed that the number of newly generated cells in the adult V-SVZ is reduced by NTN5 KO, assessed either 1 or 7 days after EdU administration. The role of NTN5 in the proliferation of cells in the neurogenic niche in the V-SVZ complements that of other guidance proteins, such as ephrin B3, EphA4, and semaphorin 3A (Furne et al., 2009). Ephrin B3 blocks caspase-dependent cell death by binding to the EphA4 receptor, and mice lacking EphA4 have large numbers of neuroblasts in the V-SVZ. Overexpression of semaphorin 3A (via knockdown of miR30c) reduces proliferation in the V-SVZ while promoting stem cell differentiation (Sun et al., 2016). We provide new evidence that NTN5 is another important contributor.

NTN5 is highly expressed in the transit-amplifying (type C) cells and neuroblasts (type A cells) in the V-SVZ (Yamagishi et al., 2015). We found a reduction in the percentage of EdU-labeled cells that were DCX positive in NTN5 KO mice, suggesting that the ratio of proliferating type C cells was increased (Figure 4D). However, as the number of EdU-labeled cells was also reduced, the actual number of total type C cells was not changed. These results suggest that differentiation of type C cells into type A cells may be slower in the mutant mice. In addition, the number of Ki67-positive cells was not changed, but the proportion that were EdU labeled was reduced; thus, the cell cycle seems to be longer in the NTN5 KO. Although the number of EdU-labeled cells was decreased in the V-SVZ, the number in the OB was not affected by NTN5 KO. Interestingly, we observed misalignment, partial disruption, and broadening of the migratory bundle in the RMS (Figures 4H–J). The disorganization of neuroblast migration also occurs with deletion of other guidance molecules such as Slit1 and EphA4, which repel astrocytes and neuroblasts (Nguyen-Ba-Charvet, 2004; Kaneko et al., 2010; Todd et al., 2017). However, unlike that in EphA4 mutant mice, a reduction of EdU-labeled cells in the OB was not observed in NTN5 mutant mice. The overall size of the OB did not change, whereas the OBs are smaller in other models with RMS migration defects, such as Girdin and F0621- and β8-integrin mutant mice (Belvindrah et al., 2007; Mobley and McCarty, 2011; Wang et al., 2011).

Garrett et al. (2016) reported that NTN5 in the developing mouse is expressed in boundary cap cells, which comprise a transient neural crest-derived population adjacent to the embryonic spinal cord that repel motor neurons to prevent aberrant migration outside the ventral horn. In NTN5 KO mice, the motor neurons abnormally enter the ventral roots and migrate into the peripheral nervous system (Garrett et al., 2016). Additionally, they found that DCC mutant mice also showed a similar phenotype, indicating that NTN5 in boundary cap cells binds to DCC expressed on motor neurons, possibly coexpressing Unc5 receptors, to repel them. NTN5 also binds to DSCAM, another netrin-1 receptor (Visser et al., 2015). However, this interaction was identified by biochemical screening, and functional analyses have not been performed.

In addition to the reduction of proliferating cells in the V-SVZ of NTN5 KO mice, we found a significant increase of newly generated Olig2-positive cells (oligodendrocyte lineage cells) (Nishiyama et al., 2009; Kuhn et al., 2019) outside this area. OPCs are distributed in both white and gray matter throughout the brain and are considered a pool of migratory and proliferative cells (Bergles and Richardson, 2015). When we administrated EdU pulse for 4 h, we observed a significant number of Olig2-positive/EdU-labeled cells in the CTX of NTN5 KOs, indicating local proliferation in the area (Supplementary Figure 2E). Interestingly, netrin-1 inhibits proliferation and promotes the differentiation of OPCs expressing DCC and Unc5A receptors (Tepavčević et al., 2014). NTN5 may similarly regulate oligodendrogenesis by binding to DCC/Unc5A. OPCs migrate along the vasculature during development and stay adjacent to vessels, maintaining the potential to differentiate into oligodendrocytes (Tsai et al., 2016). As NTN5 is also expressed by some vascular endothelial cells throughout the brain (Yamagishi et al., 2015), we surmise that these cells signal to OPCs, as we often observed pairs of EdU- and Olig2-positive cells adjacent to endothelial cells in NTN5 KO mice.

Interestingly, we observed a lower density of EdU-labeled cells in the V-SVZ of NTN5 KO mouse 1 and 7 days after EdU administration but no change in the numbers of EdU-labeled neuroblasts in the granule layer of the OB. It is possible that the newly generated neuroblasts in the V-SVZ allowed rapid migration in NTN5 KO mice or that proliferation of type A cells in the RMS was accelerated. Indeed, EdU-labeled cells were abundant in the rostral RMS of NTN5 KO mice (Supplementary Figure 6). Another possible explanation is that NTN5 acts as an adhesive/attractant for neuroblasts, hindering their migration through the RMS. When neuroblasts migrate through RMS, they need to repel astrocytes via the Slit-Robo pathway (Kaneko et al., 2010), and the loss of adhesion by NTN5 deletion may result in unopposed repulsive activities. Further studies are needed to resolve this discrepancy between reduced proliferation in the neurogenic niche and normal neuroblast numbers in the granule layer of the OB. Nevertheless, NTN5 may play a role as an adhesive molecule that holds neuroblasts in a chain, as this organization was disturbed in the mutant mice (Figures 4H–J).

Of note, the increase in OPCs observed in NTN5 KO mice suggests a potentially new avenue of study toward clinical approaches for demyelinating diseases such as multiple sclerosis. Multiple sclerosis is a chronic, inflammatory, demyelinating, and neurodegenerative disease caused by the infiltration of inflammatory cells into the central nervous system (Filippi et al., 2018). These cells induce oligodendrocyte and neuro-axonal damage through cell contact, the secretion of soluble mediators, and oxidative stress. In multiple sclerosis lesions, the differentiation of OPCs into oligodendrocytes is impaired, impeding efficient remyelination (Kuhlmann et al., 2008). As newly differentiated oligodendrocytes are observed in successfully remyelinated lesions, the role of NTN5 as a regulator of oligodendrogenesis may hold therapeutic promise.

## Conclusion

**FIGURE 5:**
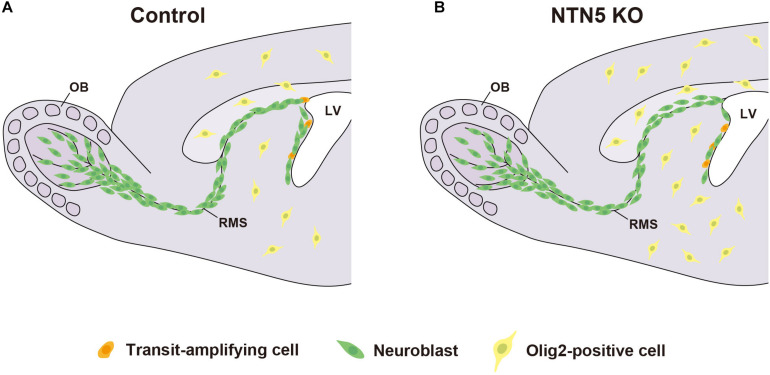
Model for neurogenesis and oligodendrogenesis in the adult brain. Schematic drawing of control **(A)** and NTN5 KO **(B)** mouse brains. The proliferation in the neurogenic niche is decreased, whereas the generation of oligodendrocyte lineage cells outside the V-SVZ is increased in NTN5 KO mice. Neuroblasts are misaligned in the RMS. Nevertheless, the number of neuroblasts migrating to the OB is not different.

In conclusion, our findings suggest that NTN5 directly or indirectly promotes proliferation in the neurogenic niche in the adult V-SVZ and inhibits oligodendrogenesis throughout the brain (Figure 5). However, *in vitro* analyses are needed to clarify the intracellular signaling triggered by NTN5, such as binding assays to define receptors as well as turning and growth cone collapse assays. These studies will lead to a better understanding of the molecular mechanisms by which NTN5 regulates proliferation in the neurogenic niche and oligodendrogenesis.

## Data Availability Statement

The original contributions presented in the study are included in the article/Supplementary Material, further inquiries can be directed to the corresponding author.

## Ethics Statement

The animal study was reviewed and approved by the Animal Research Committee of Hamamatsu University School of Medicine and those of Niigata University.

## Author Contributions

SI and SY designed, analyzed, and wrote the manuscript. SI, YI, SM, and SY performed the research. LZ, MA, and KeS generated NTN5 KO mice. KoS analyzed the data. All authors have seen and agreed with the content of the manuscript.

## Conflict of Interest

The authors declare that the research was conducted in the absence of any commercial or financial relationships that could be construed as a potential conflict of interest.
