# Supplementary material for: Decreased Proliferation in the Neurogenic Niche, Disorganized Neuroblast Migration, and Increased Oligodendrogenesis in Adult Netrin-5-Deficient Mice
Source: Front Neurosci. 2020 Nov 26;14:570974. doi: 10.3389/fnins.2020.570974 (PMC7726356; doi:10.3389/fnins.2020.570974)
Supplement: Supplementary file 1 [file Data_Sheet_1.pdf]

## *Supplementary Material*

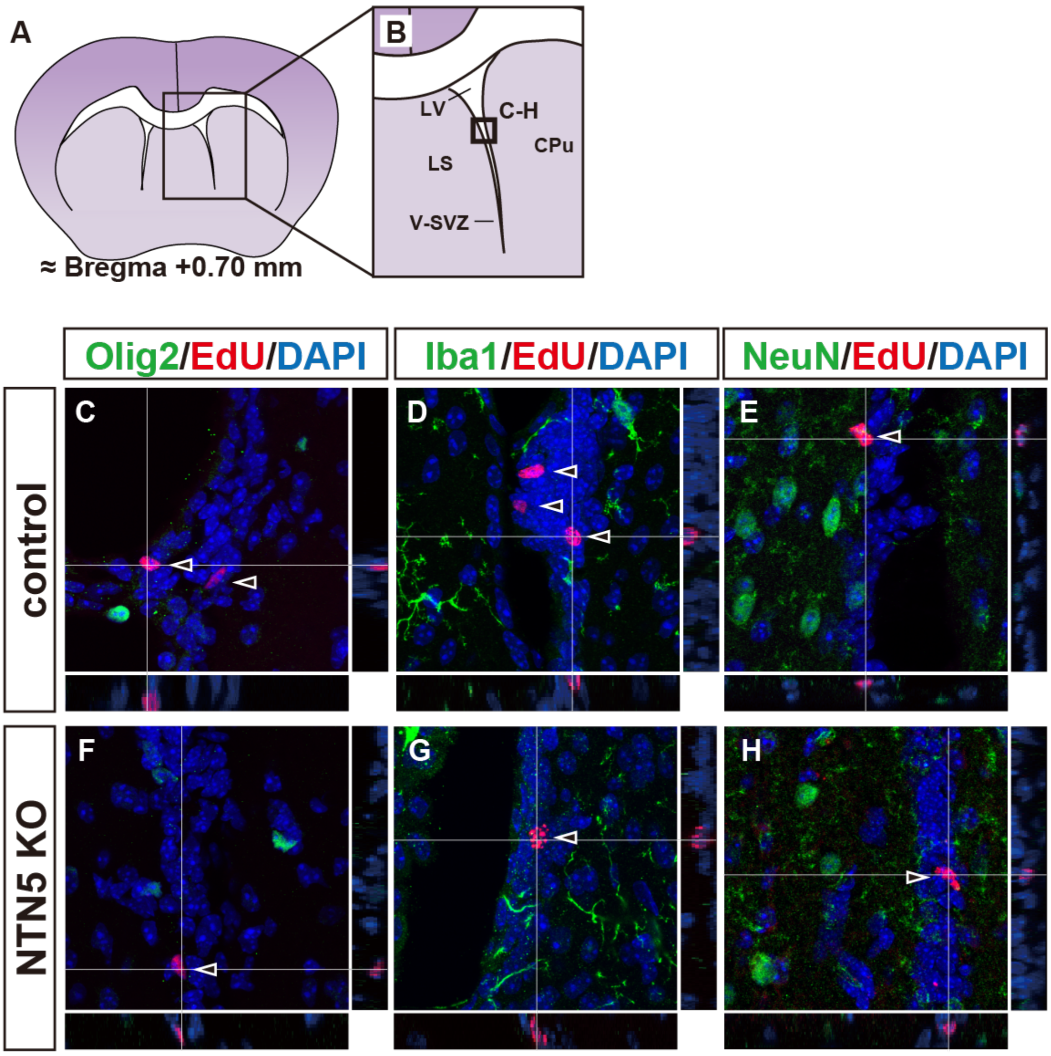


**Supplementary FIGURE 1 | EdU-labeled cells in the V-SVZ are not positive for Olig2, Iba1, or NeuN. (A, B)** Schematic drawing of a coronal section of adult mouse brain at bregma + 0.70 mm. **(B)** Enlargement of boxed region shown in panel **A**. The square box indicates the area observed for immunostaining. **(C, F)** Representative confocal images with orthogonal views for Olig2 (green), EdU (red), and DAPI (blue) 7 days after EdU administration. EdU-labeled cells were Olig2 negative (black arrowheads). **(D, G)** Representative confocal images with orthogonal views for Iba1 (green), EdU (red), and DAPI (blue) 7 days after EdU administration. EdU-labeled cells were Iba1 negative (black arrowheads). **(E, H)** Representative confocal images with orthogonal views for NeuN (green), EdU (red), and DAPI (blue) 7 days after EdU administration. EdU-labeled cells were NeuN negative (black arrowheads). CPu, caudate putamen; LS, lateral septal nucleus; LV, lateral ventricle; V-SVZ, ventricular-subventricular zone.

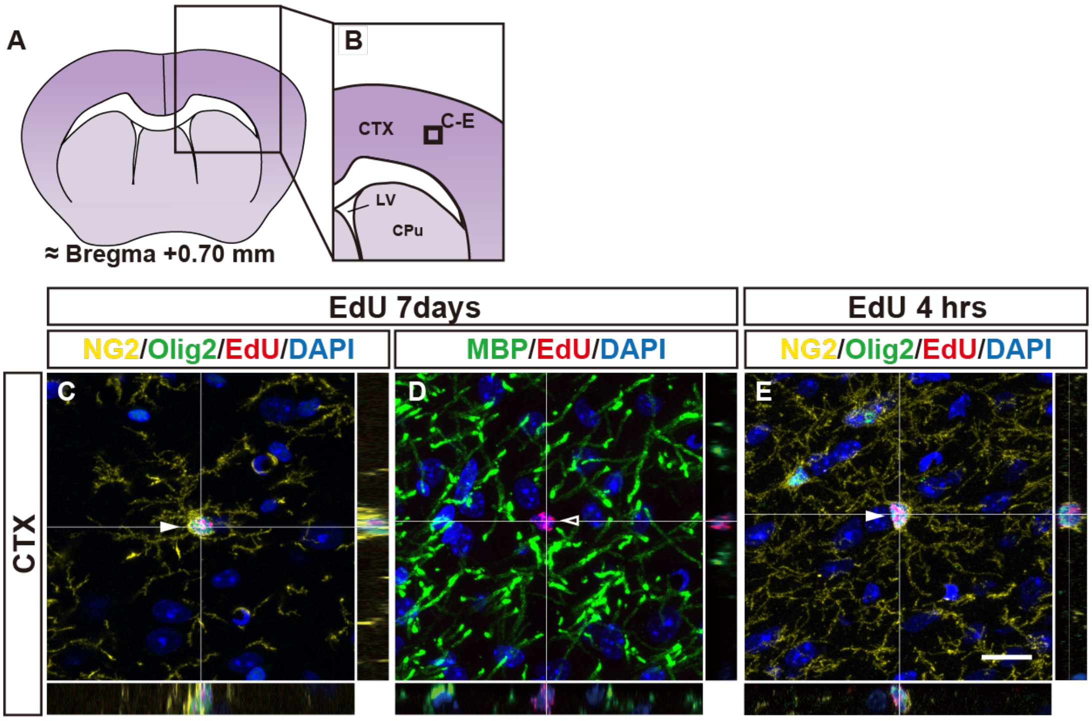


**Supplementary FIGURE 2 | Oligodendrogenesis in the cortex of NTN5 KO mice. (A, B)** Schematic drawing of a coronal section of adult mouse brain at bregma + 0.70 mm. **(B)** Enlargement of boxed region shown in panel **A**. The square box indicates the area observed for immunostaining. **(C)** Representative confocal image with orthogonal views of the CTX for NG2 (yellow), Olig2 (green), EdU (red), and DAPI (blue) 7 days after EdU administration. Almost all EdU- and Olig2-positive cells in the CTX were NG2 positive (white arrowhead). **(D)** Representative confocal image with orthogonal views of the CTX for MBP (green), EdU (red), and DAPI (blue) 7 days after EdU administration. None of EdU-labeled cells in the CTX were MBP positive (black arrowhead). **(E)** Representative confocal image with orthogonal views of the CTX for NG2 (yellow), Olig2 (green), EdU (red), and DAPI (blue) 4 h after EdU administration. Scale bar indicates 15 µm. CTX, cerebral cortex; CPu, caudate putamen; LV, lateral ventricle.

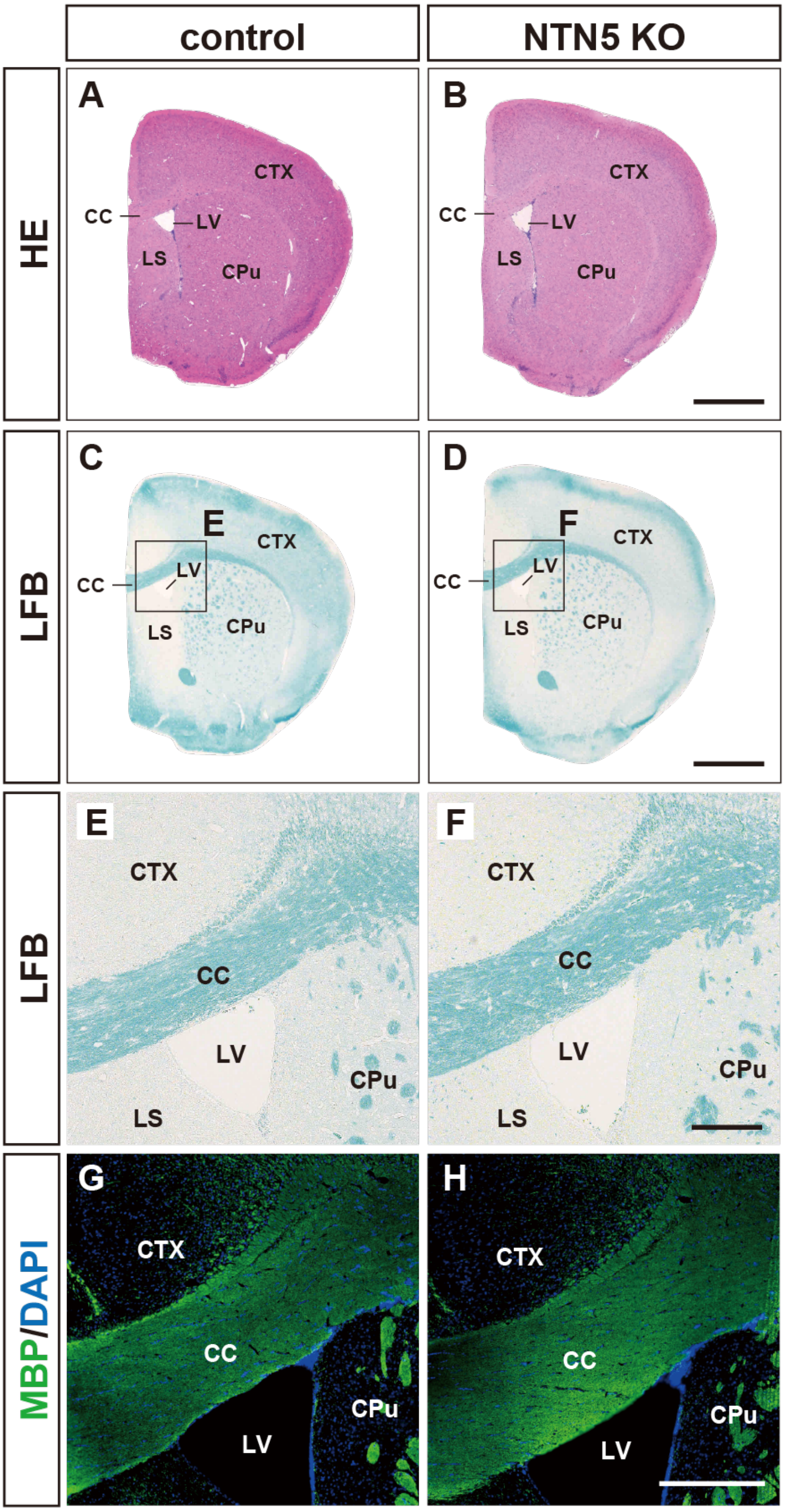


**Supplementary FIGURE 3 | Normal morphological features of adult NTN5 KO mouse brain. (A, B)** HE staining of a coronal section of adult brain. **(C, D)** LFB staining of a coronal section of adult brain. **(E, F)** Enlargements of boxed regions shown in panels **C** and **D**, respectively. **(G, H)** Fluorescence images of CC for MBP (green) and DAPI (blue). Scale bars indicate 1 mm (**A–D**) and 200 μm (**E–H**). CC, corpus callosum; CTX, cerebral cortex; CPu, caudate putamen; LV, lateral ventricle.

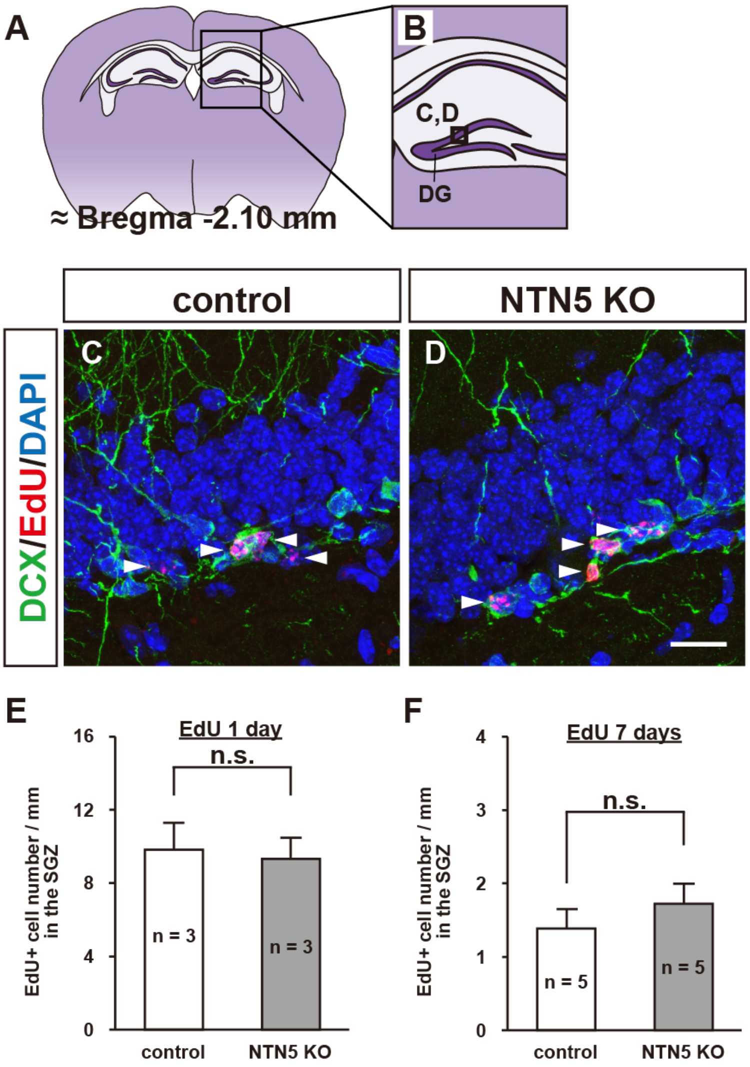


**Supplementary FIGURE 4 | Normal number of EdU-labeled cells in the SGZ of the hippocampal DG in adult NTN5 KO mice 1 day and 7 days after EdU injection. (A, B)** Schematic drawing of a coronal section of adult mouse brain at bregma – 2.10 mm. **(B)** Enlargement of boxed region shown in panel **A**. The square box indicates the area observed for immunostaining. **(C, D)** Representative confocal images with orthogonal views of the SGZ 1 day after EdU administration for DCX (green), EdU (red), and DAPI (blue). More than half of the EdU-labeled cells observed in the SGZ of both control and NTN5 KO mice were DCX positive (white arrowheads). **(E, F)** Quantification of EdU-labeled cells 1 day and 7 days after EdU administration (1 day and 7 days, $n = 3$ and $n = 5$, respectively, for both genotypes). Scale bar indicates 15 µm. Data are means ± SEs. n.s., not significant. DG, dentate gyrus; SGZ, subgranular zone.

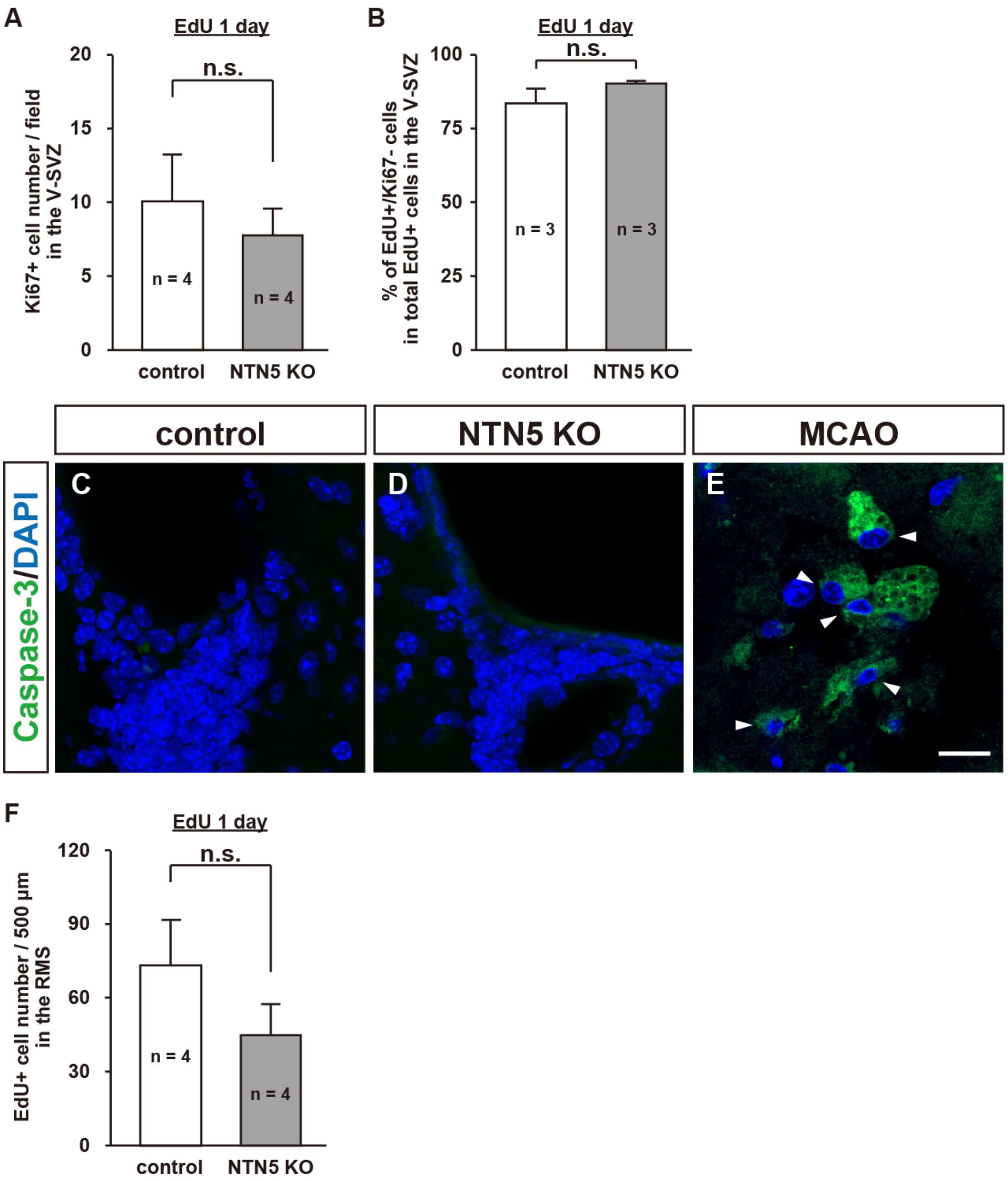


**Supplementary FIGURE 5 | Analysis of cell cycle in the V-SVZ. (A)** Quantification of Ki67-positive cells 1 day after EdU administration. Three fields per animal were analyzed (*n* = 4 for both genotypes). **(B)** Quantification of Ki67-negative cells among EdU-labeled cells (cell cycle exit index). Three fields per animal were analyzed (*n* = 3 for both genotypes). **(C, D)** Representative confocal images of the V-SVZ and the CTX for active caspase-3 (green) and DAPI (blue). No active caspase-3-positive cells were observed in the V-SVZ of control or NTN5 KO mice. **(E)** Positive control for anti-active caspase-3 antibody staining observed in the peri-infarct region 3 days after middle cerebral artery occlusion (MCAO) (white arrowheads). **(F)** Quantification of EdU-labeled cells in the RMS; 500 µm per animal were analyzed (*n* = 4 for both genotypes). Scale bar indicates 15 µm. Data are means ± SEs. n.s., not significant. RMS, rostral migratory stream; V-SVZ, ventricular-subventricular zone.

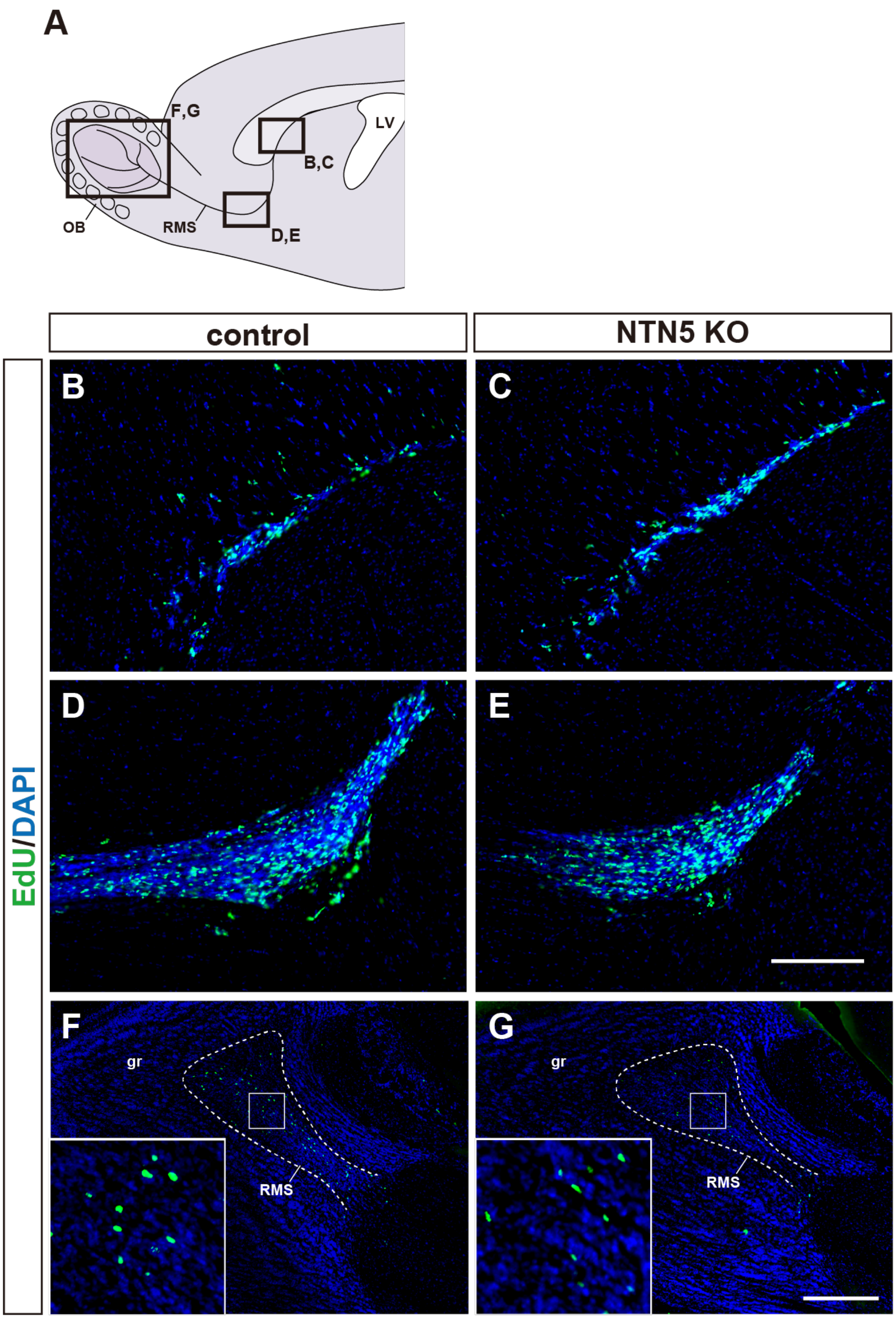


**Supplementary FIGURE 6 | Numbers of EdU-labeled cells are normal from the rostral RMS to the OB in NTN5 KO mice. (A)** Schematic drawing of a sagittal section. **(B–G)** DAPI (blue) and EdU (green) labeling in the brains of adult control and NTN5 KO mice 1 day after EdU administration. Note that EdU-labeled cells migrated similarly in control and NTN5 KO mice at the rostral part of the RMS. Insets show higher magnification of boxed areas **(F, G)**. Scale bars indicate 200 µm (**B–E**) and 400 µm (**F, G**). LV, lateral ventricle; RMS, rostral migratory stream.
